# Supplementary material for: Activation of the glutamatergic cingulate cortical-cortical connection facilitates pain in adult mice
Source: Commun Biol. 2023 Dec 9;6:1247. doi: 10.1038/s42003-023-05589-1 (PMC10710420; doi:10.1038/s42003-023-05589-1)
Supplement: Supplementary file 4 — Reporting Summary [file 42003_2023_5589_MOESM4_ESM.pdf]

nature portfolio

Corresponding author(s): Min Zhuo

Last updated by author(s): Oct 11, 2023

Reporting Summary

Nature Portfolio wishes to improve the reproducibility of the work that we publish. This form provides structure for consistency and transparency in reporting. For further information on Nature Portfolio policies, see our Editorial Policies and the Editorial Policy Checklist.

Statistics

For all statistical analyses, confirm that the following items are present in the figure legend, table legend, main text, or Methods section.

n/a	Confirmed	
☐	☒	The exact sample size (n) for each experimental group/condition, given as a discrete number and unit of measurement
☐	☒	A statement on whether measurements were taken from distinct samples or whether the same sample was measured repeatedly
☐	☒	The statistical test(s) used AND whether they are one- or two-sided<br>*Only common tests should be described solely by name; describe more complex techniques in the Methods section.*
☒	☐	A description of all covariates tested
☐	☒	A description of any assumptions or corrections, such as tests of normality and adjustment for multiple comparisons
☐	☒	A full description of the statistical parameters including central tendency (e.g. means) or other basic estimates (e.g. regression coefficient) AND variation (e.g. standard deviation) or associated estimates of uncertainty (e.g. confidence intervals)
☐	☒	For null hypothesis testing, the test statistic (e.g. F, t, r) with confidence intervals, effect sizes, degrees of freedom and P value noted<br>*Give P values as exact values whenever suitable.*
☒	☐	For Bayesian analysis, information on the choice of priors and Markov chain Monte Carlo settings
☒	☐	For hierarchical and complex designs, identification of the appropriate level for tests and full reporting of outcomes
☒	☐	Estimates of effect sizes (e.g. Cohen's d, Pearson's r), indicating how they were calculated

Our web collection on statistics for biologists contains articles on many of the points above.

Software and code

Policy information about availability of computer code

Data collection	For slice physiological recording, the current and voltage signals were recorded with Axon 200B amplifier (Molecular Devices) and Clampex 10 data acquisition software (Molecular Devices). Images were captured on a Olympus confocal microscope (FV1000, Olympus, Japan) or a slide scanner (Slideview VS200, Olympus).
Data analysis	For slice physiological recording, the data was analyzed using Clampfit 10.2 (Molecular devices). OriginPro 8.0 software (Origin Lab Corporation, USA) and GraphPad Prism 8.0 (Graph Pad Software, Inc., USA) were used for the statistical analyses.

For manuscripts utilizing custom algorithms or software that are central to the research but not yet described in published literature, software must be made available to editors and reviewers. We strongly encourage code deposition in a community repository (e.g. GitHub). See the Nature Portfolio guidelines for submitting code & software for further information.

Data

Policy information about availability of data

All manuscripts must include a data availability statement. This statement should provide the following information, where applicable:

- Accession codes, unique identifiers, or web links for publicly available datasets
- A description of any restrictions on data availability
- For clinical datasets or third party data, please ensure that the statement adheres to our policy

The data that support the findings of this study are available from the corresponding author upon reasonable request.

Research involving human participants, their data, or biological material

Policy information about studies with human participants or human data. See also policy information about sex, gender (identity/presentation), and sexual orientation and race, ethnicity and racism.

Reporting on sex and gender	Not Applicable
Reporting on race, ethnicity, or other socially relevant groupings	Not Applicable
Population characteristics	Not Applicable
Recruitment	Not Applicable
Ethics oversight	Not Applicable

Note that full information on the approval of the study protocol must also be provided in the manuscript.

Field-specific reporting

Please select the one below that is the best fit for your research. If you are not sure, read the appropriate sections before making your selection.

☒ Life sciences ☐ Behavioural & social sciences ☐ Ecological, evolutionary & environmental sciences

For a reference copy of the document with all sections, see nature.com/documents/nr-reporting-summary-flat.pdf

Life sciences study design

All studies must disclose on these points even when the disclosure is negative.

Sample size	No statistical methods were used to pre-determine sample sizes, but sample sizes for this study are similar to those reported in previous publications (PMIDs: 34289348, 35443154)
Data exclusions	For all experiments, mice with signs of infection/bleeding/unhealthy conditions after the surgeries were excluded for behavioral tests and mice with missed viral injections targets, as described by brain atlas, were not included in experimental analyses.
Replication	Behavioral experiments are replicated multiples times with independent mice. Numbers of replicates (n) are indicated in the figure legends.
Randomization	The animals in the behavioral tests were randomized assigned.
Blinding	All investigators were blinded to group allocation during data collection and analysis.

Reporting for specific materials, systems and methods

We require information from authors about some types of materials, experimental systems and methods used in many studies. Here, indicate whether each material, system or method listed is relevant to your study. If you are not sure if a list item applies to your research, read the appropriate section before selecting a response.

Materials & experimental systems

n/a	Involved in the study	
☒	☐	Antibodies
☒	☐	Eukaryotic cell lines
☒	☐	Palaeontology and archaeology
☐	☒	Animals and other organisms
☒	☐	Clinical data
☒	☐	Dual use research of concern
☒	☐	Plants

Methods

n/a	Involved in the study	
☒	☐	ChIP-seq
☒	☐	Flow cytometry
☒	☐	MRI-based neuroimaging

Animals and other research organisms

Policy information about studies involving animals; ARRIVE guidelines recommended for reporting animal research, and Sex and Gender in Research

Laboratory animals	Adult (8 to 12 weeks old) male or female C57BL/6 mice were purchased from the Experimental Animal Center of Xi'an Jiaotong University. All animals were randomly housed under an artificial 12-hour light/dark cycle (7 a.m. to 7 p.m. light) in a holding room kept at a temperature of 23 ± 1°C with food and water provided ad libitum.
Wild animals	The study did not involve wild animals.
Reporting on sex	Both male or female C57BL/6 mice were used in the study.
Field-collected samples	This study did not involve samples collected from the field.
Ethics oversight	All experiment protocols have been approved by the Ethics Committee of Xi'an Jiaotong University.

Note that full information on the approval of the study protocol must also be provided in the manuscript.

Plants

Seed stocks	Not Applicable
Novel plant genotypes	Not Applicable
Authentication	Not Applicable
